# Trends in Changes of Automatic Milking System Biomarkers and Their Relations with Blood Biochemical Parameters in Fresh Dairy Cows

**DOI:** 10.3390/vetsci8030045

**Published:** 2021-03-09

**Authors:** Dovilė Malašauskienė, Ramūnas Antanaitis, Vida Juozaitiene, Mindaugas Televičius, Mingaudas Urbutis, Arūnas Rutkauskas, Agnė Šimkutė, Giedrius Palubinskas

**Affiliations:** 1 Large Animal Clinic, Veterinary Academy, Lithuanian University of Health Sciences, Tilžės 18, LT-47181 Kaunas, Lithuania; mindaugas.televicius@lsmuni.lt (M.T.); mingaudas.urbutis@lsmuni.lt (M.U.); arunas.rutkauskas@lsmuni.lt (A.R.); 2 Department of Animal Breeding, Veterinary Academy, Lithuanian University of Health Sciences, Tilžės 18, LT-47181 Kaunas, Lithuania; vida.juozaitiene@lsmuni.lt (V.J.); giedrius.palubinskas@lsmuni.lt (G.P.); 3 Veterinary Academy, Lithuanian University of Health Sciences, Tilžės 18, LT-47181 Kaunas, Lithuania; agne.simkute@stud.lsmu.lt

**Keywords:** NEFAs, dairy cows, cortisol, fat mobilization

## Abstract

The aim or this study was to determine the relationship between non-esterified fatty acids and biomarkers from an automatic milking system (AMS). Fresh dairy cows (*n* = 102) were selected and milked in Lely Astronaut^®^ A3 milking robots. The rumination time (RT), body weight (BW), milk content and composition parameters, milk fat/protein ratio (F/P), and milk electrical conductivity were registered by the same milking robots. For examining non-esterified fatty acids (NEFAs), blood samples were acquired from cows in the dry period. According to the NEFA concentrations, all cows were divided into two groups: Group I, with <0.300 mEq/L (*n* = 66), and Group II, with ≥0.300 mEq/L (*n* = 36). Albumin (ALB), aspartate aminotransferase (AST), gamma-glutamyl transferase (GGT), and cortisol concentrations were also analyzed once a week up to 30 days in milking. The study revealed that the cows in Group I had higher concentrations of ALB, cortisol, and GGT, but the average concentration of AST was lower. In Group 1, the milk F/P was higher, but the milk yield was lower. We hypothesize that biomarkers from AMS could help in the early diagnosis of metabolic diseases after calving or to control negative energy balance before calving.

## 1. Introduction

In recent years, a wide variety of devices have been developed and implemented by the dairy industry to aid in monitoring the physiological parameters and behavior of cows [1]. Cows with health disorders are expected to demonstrate changes of sufficient magnitude in their activity and rumination behavior to be detected by specially designed algorithms [2]. Changes in activity and rumination time can be associated with subclinical and clinical health disorders [2,3]. According to our past study, some biomarkers from automatic milking systems (AMSs), like milk yield (MY), rumination time (RT), body weight (BW), milk composition, milk conductivity, and their relationship with some blood parameters, have an association with subclinical ketosis (SCRTK) and subclinical acidosis (SARA) [4]. Juozaitiene et al. [5] found that changes in cows’ AMS indicators can act as an additional tool to improve the management of reproduction in dairy herds, but more research-based studies are needed to incorporate them into practice. Malasauskiene et al. [6] established that RT could be used as an indicator of stress for cows in the first 30 days postpartum. Rumination time, subsequent yield, and milk trait changes depend on the dairy cow’s lactation period and reproduction status [7]. Changes in activity and rumination indicate specific patterns for specific disorders. Still, there is a lack of research on the performance of the automatic health monitoring system (AHMS) that uses these data to identify animals with digestive and metabolic disorders [7]. More data and research are also needed about the specific patterns of activity and rumination, particularly when a clinical diagnosis (CD) of digestive and metabolic disorders is carried out [2].

Most of the time, transition dairy cows are in a state of negative energy balance (NEB) for three main reasons: growing energy requirements for parturition, a drop in dry matter intake (DMI) around calving, and a shortage of DMI compared with the energy demand for milk production [8]. The concentration of blood plasma non-esterified fatty acids (NEFAs) was used as a biomarker to describe energy status (ES). At the beginning of lactation, the dry matter intake of high-production cows rarely fulfills their energy requirements [9]. To make up for insufficient energy, cows mobilize energy from their body reserves. Even though a negative ES is very often present for today’s high-production cows for a couple of weeks in early lactation, a major and prolonged negative ES usually causes reproduction and health problems [10]. Some degree of NEB, which presents as increased concentrations of NEFAs and beta-hydroxybutyrate (BHB), is to be expected in the transition period as the cows become accustomed to the growing energy demand while energy uptake for adequate production reaches sufficient levels [11]. With the decrease in blood glucose concentration around parturition, NEFA mobilization from adipose tissue is activated, and an increase in fatty acid uptake by the liver is noted [12]. Accompanying the decrease in glucose concentration, naturally caused by a lack of insulin, an increase in glucagon secretion is seen. This takes part in the transportation of NEFAs into the mitochondria, which, in turn, increases the ketone body formation [13]. Marginally increased rates of lipid mobilization result in excessive uptake of NEFAs by the liver and increased triglyceride accumulation, which is followed by the development of hepatic lipidosis [12]. Severe NEB has detrimental effects on dairy cows’ health and production because it is suspected that energy deficiency relates to immunosuppression [14,15,16]. According to Ospina et al. [11], the ability to identify, at the cow level, animals that are more prone to developing a disease based on BHB and NEFA concentrations would allow producers to prevent diseases proactively by concentrating their efforts on nutritional and management strategies. It is evident that biomarkers and blood parameters can be used in early disease diagnostics, but there is limited information about the relation between the blood metabolic profile and some biomarkers from automated health-monitoring systems in the literature. Therefore, the current study aimed to determine the relationship between non-esterified fatty acids and biomarkers from an automatic milking system in fresh dairy cows.

## 2. Materials and Methods

### 2.1. Location, Animals, and Experimental Design

This study followed the provisions of the Law on Animal Welfare and Protection of the Republic of Lithuania (Official Gazette Valstybes žinios, 1997, No. 108-2728; 2012, No. 122-6126), 22 September Directive 2010/63/EU of the European Parliament, and of the Council on the protection of animals used for scientific purposes (OJ 2010 L 276, p. 33) with regard to the European Convention for the protection of vertebrate animals used for experimental and other scientific purposes (Official Journal 2007, No 49-1883, No. 49-1884). The study approval number was PK016965.

The study was conducted in the period of 1 September 2019 to 31 December 2019. The experiment took place on a dairy farm in the central region of Europe at 56°00′ N, 24°00′ E. Lithuanian Black and White dairy cows (*n* = 102) in their dry period were selected according to the criteria of having had two or more lactations (on average 2.6 ± 0.08 lactations) and being clinically healthy. Two to three weeks prior to the planned parturition date, the blood NEFA concentrations were evaluated for these cows, and according to the results they were divided into two groups: Group I, with NEFA levels of <0.300 mEq/L (*n* = 66), and Group II, with NEFA levels of ≥0.300 mEq/L (*n* = 36) [15]. After parturition, each cow was monitored from Day 1 until Day 30 in milk. Blood samples were taken weekly, and daily data from the automated milking systems were gathered. According to the data of the milk fat-to-protein ratio (F/P), the cows were grouped into three levels: F/P < 1 (indicating subclinical acidosis), F/P 1–1.23 (healthy cows), and F/P > 1.24 (indicating subclinical ketosis) [16].

A loose housing system was provided for the cows, and they received a total mixed ration (TMR) throughout the year at regular times, balanced to meet their physiological requirements. The TMR consisted of 50% grain concentrate mash, 10% grass silage, 30% corn silage, and 4% grass hay. The diets followed the recommendations of NRC (2001) [17] to provide adequate nutrients for a 550 kg Holstein cow producing 35 kg of milk per day. Cow feeding took place every day at 06:00 and 18:00. The research started on the fifth day and continued until the 30th day after calving.

### 2.2. Measurements

The cows were milked in Lely Astronaut^®^ A3 milking robots (Lely, Maassluis, The Netherlands) with free traffic. To motivate the cows to visit the robot, they received 2 kg of concentrates per day while inside the milking robot. Rumination time (RT), milk protein (MP), milk fat (MF), body weight (BW), milk yield (MY), milk lactose (ML), milk fat/protein ratio (F/P), milk somatic cell count (SCC), and milk electric conductivity of all quarters of the udder (front left (EC1), front right (EC2), rear left (EC3), rear right (EC4)) were registered with the help of the Lely Astronaut^®^ A3 milking robots and TC4 management system.

Blood sampling took place at 10:00 a.m., before the afternoon feeding. A volume of 10 mL of blood from the coccygeal vein was collected into a basic evacuated red-top tube (BD Vacutainer, Crawley, UK). The samples were transported for analysis at the Large Animal Clinic’s Laboratory of Clinical Tests at the Veterinary Academy of the Lithuanian University of Health Sciences. For the examination of NEFAs, blood samples were acquired from cows in the dry period (2–3 weeks before calving). All samples of NEFAs were analyzed using an automated wet chemistry analyzer (Rx Daytona, Randox Laboratories Ltd., London, UK) with specific reagents (Rx Daytona, Randox Laboratories Ltd., London, UK).

Blood samples for the analysis of other parameters were taken soon after calving, once per week until 30 days in milk. The obtained blood serum was examined using a Hitachi 705 analyzer (Hitachi, Tokyo, Japan) and DiaSys reagents (Diagnostic Systems GmbH, Dusseldorf, Germany). The following parameters and their concentrations were determined: albumin (Alb), aspartate aminotransferase (AST), and gamma-glutamyltransferase (GGT). The blood samples were analyzed for cortisol concentrations using the fluorescence enzyme immunoassay method with a Tosoh Corporation AIA-360 (South San Francisco, CA, USA).

### 2.3. Data Analysis and Statistics

The SPSS 25.0 (SPSS Inc., Chicago, IL, USA) program package was used for statistical analysis of the data. The normality of the distributions of the evaluated traits was assessed using the Kolmogorov–Smirnov test. The results are provided as the mean plus or minus the standard error of the mean (*M* ± *SE*). The linear Pearson correlation (*r*) was calculated to evaluate the statistical relationship between NEFAs and the following biomarkers from the automated health-monitoring system in fresh dairy cows: BW, MY, MF, MP, ML, SCC, electrical conductivity of milk at the udder quarters level (left rear, right rear, left front, right front), RT, and blood indicators (ALB, AST, GGT, and cortisol). To analyze the SCC, it was log transformed to log_10_ SCC. Differences in the mean values of normally distributed variables were analyzed using Student’s *t*-test. A probability of less than 0.05 was considered reliable (*p*-value < 0.05).

The data were grouped by the NEFA level of cows and by class of F/P in milk (HL, healthy cows (*n* = 51); SCA, subclinical acidosis (*n* = 42); SCK, subclinical ketosis (*n* = 9)) [18]. The relationship between class according to milk F/P and group of cows according to NEFA level was evaluated using Pearson’s χ^2^ test.

## 3. Results

### 3.1. The Blood Metabolic Profile (NEFAs and Other Blood Biochemical Parameters) in Fresh Dairy Cows

Our study revealed that the average concentrations of ALB, cortisol, and GGT were higher in Group I than in Group II. The AST concentration of Group I was lower than that of Group II (Table 1).

### 3.2. Relations between the Blood Metabolic Profile and Biomarkers from the Automated Health-Monitoring System in Fresh Dairy Cows

The milk yield and milk protein content of the cows (Table 2) were statistically different between both groups and depended on the level of NEFAs (*p <* 0.01). The MY was lower in Group I compared to Group II, while the milk fat/protein ratio (MF/MP) was higher in Group I than in Group II.

**Figure 1:**
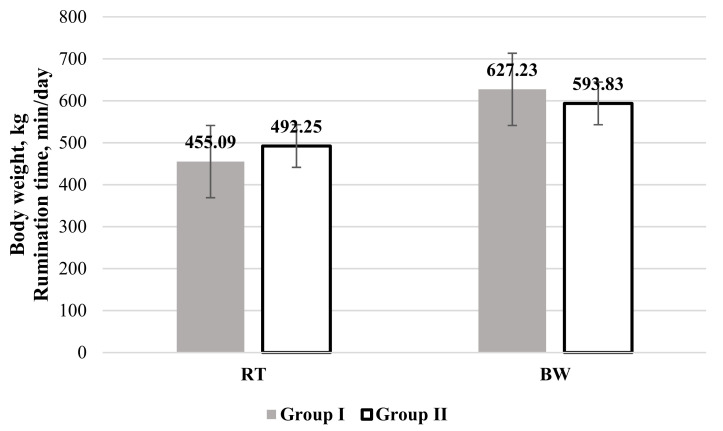
Rumination time (RT) and body weight (BW) in cows categorized by their non-esterified fatty acids (NEFAs) level. Group I: NEFA < 0.300 mEq/L; Group II: NEFA ≥ 0.300 mEq/L.

Analysis of the RT (Figure 1) revealed that values of this indicator were higher in Group II, but this was statistically unreliable.

### 3.3. Correlations between Blood Metabolic Profile Parameters and Some Automated Milking System Biomarkers

**Figure 2:**
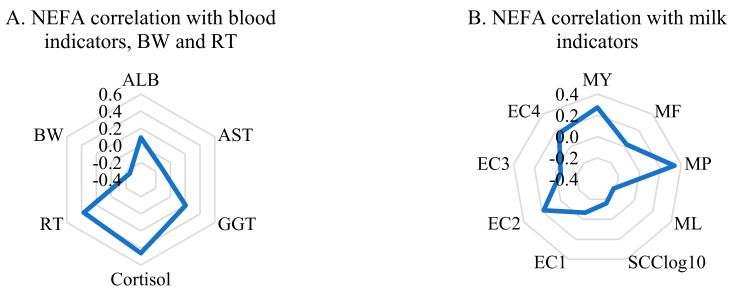
Correlations of NEFA levels with (**A**) blood indicators and (**B**) biomarkers from the automated health-monitoring system.

Correlation analysis showed a positive relationship of NEFA values with blood cortisol (*r* = 0.460, *p* < 0.01). There was also a significant positive correlation between RT and NEFA values (*r* = 0.369, *p* < 0.01) and a negative correlation between NEFA values and the BW of cows (*r* = −0.254, *p* < 0.05). Blood ALB and GGT values were slightly positively related to NEFA levels; however, AST and NEFA correlated in opposite directions (Figure 2A).

The correlations between NEFA and milk traits are shown in Figure 2B. The MY (*r* = 0.274, *p* < 0.05) was positively associated with NEFA levels, as well as values of MP, but this was statistically unreliable. Correlation coefficients with other indicators were statistically unreliable.

**Figure 3:**
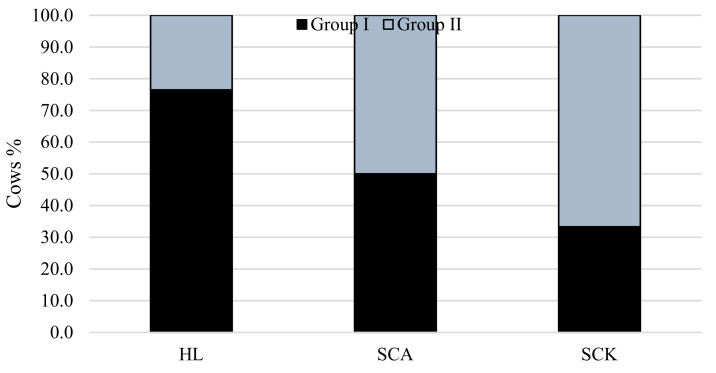
Relationship of NEFA level with the prevalence of SCA and SCK in cows. χ^2^ = 41.9, *df* = 2, *p* < 0.001. HL: healthy cows; SCA: subclinical acidosis; SCK: subclinical ketosis.

Figure 3 shows the distribution of cows from Groups I and II in the classes of different diseases and healthy cows according to milk F/P. Cows from Group II (higher NEFA levels) were more frequently diagnosed with SCK than cows from Group I. The majority of the healthy cows were cows from Group I.

## 4. Discussion

A wide variety of endocrine and metabolic blood and milk traits relate to energy balance (EB) [19,20]. Poor nutritional management and stressors reduce voluntary dry matter intake (DMI), resulting in large increases in NEFA concentrations around calving [21]. In ruminants, the central blood parameters of lipomobilization are beta-hydroxybutyrate (BHB), the most notable and abundant ketone body, and NEFAs [22]. NEFAs greatly accumulate in the liver as triglycerides (TGs), with decreased synthesis of very-low-density lipoproteins (VLDL) in the hepatocytes [20]. Our results indicate that ALB, cortisol, and GGT concentrations were higher in Group I, but AST concentrations were lower. Fatty liver is a frequent disorder at the beginning of lactation, especially in high-production dairy cattle [23]. It relates to excessive mobilization of adipose tissue fat to the liver in obese, well-conditioned cows. The negative energy balance induces this mobilization of fat during the parturient period [24]. For these cows, usually at least one of the following diseases is present: milk fever, mastitis, displaced abomasum, ketosis, metritis, or retained placenta. Low- or moderate-severity liver lipidosis may result in adequate liver functionality that does not present itself with hepatocyte destruction and shows normal activity of serum enzymes specific to the liver [20]. Even though AST activity in the serum closely correlates with liver lipidosis, this enzyme is not a specific indicator of damaged hepatocytes [25], unlike GGT, which is more specific and sensitive to liver tissue damage [26]. Infiltration of fat in the liver and subsequent breakdown of hepatocytes induces an increase in the circulation activity of cytoplasm enzymes (AST, GGT) [21]. Therefore, an increase in GGT may be due to severe fat infiltration. It has been stated that high levels of NEFA could result from a continuous decrease in blood glucose, leading to the mobilization of body fat [27]. Nonetheless, endogenous liver synthesis decreases when steatosis occurs, leading to further reduced blood glucose, cholesterol, total proteins, albumins and globulins, urea, and TGs [20].

Increased quantities of NEFAs directly leading to hepatic injury are indicated by higher plasma concentrations of GGT and AST [28]. According to Yang et al. [28], increased serum GGT could act as a marker of oxidative stress, which is strongly associated with hypertension, abnormal glucose tolerance, and dyslipidemia. Higher levels of circulating NEFAs have been related to a drop in DMI, which results in a negative protein and energy balance that lasts for at least the first weeks of lactation. Moreover, during this period, compromised immune function that predisposes several metabolic disorders, such as fatty liver, ketosis, displacement of abomasum, reproductive failure, and infectious disease, is typical [29].

Our study found that cows with higher blood NEFA concentrations had a 57.14% higher cortisol concentration. In a study by Karagiannides et al. (2014), they found that blood serum insulin levels were clearly lower in stressed rats compared to the control group and potentially contributed to elevated NEFA levels [30]. A negative energy balance, marked by increased NEFA concentrations, is considered an exceptionally stressful state for high-yielding dairy cows, as the body systems are undergoing major energy and nutrient deficiencies in the postpartum period [31].

The milk yield and milk protein content of the cows were statistically different between both groups and depended on the level of NEFAs. The MY in Group I was 10.78% lower, while F/P (8.33%) was higher than those for Group II.

A plasma NEFA concentration higher than 0.6 mmol/L at the start of lactation is often considered a base value for severe negative ES and indicates that such an animal is at greater risk of developing metabolic disorders [11]. Milk fat, lactose, and protein contents, or their ratios, were used in predicting energy status in some studies [32,33]. The results indicated that the milk fat/protein ratio, milk fat and protein contents, or fat/lactose ratio accounted for 29.1% to 31.2% of the predicted variation of the energy balance left for a cow after milk production maintenance [34]. The milk fat/protein ratio was the most informative trait in predicting ES [34]. An interaction between parity and herd concerning plasma NEFA concentrations was found in their data, probably resulting from differences in management and feeding schemes between the selected herds [34].

There was also a significant positive correlation between RT and NEFA values and a negative correlation between NEFA values and the BW of the cows in this study. Results from other studies indicate that cows in negative energy balance, sick with subclinical ketosis, tend to present decreased rumination time compared to healthy cows. However, cows diagnosed with subclinical ketosis and other postpartum diseases (e.g., mastitis, metritis, etc.) had a longer rumination time compared to healthy cows [35]. Nonetheless, from our data, we can see that cows in Group II, who had higher NEFA concentrations, also had a higher milk yield. Studies done by others have revealed a direct positive correlation between RT and milk yield [36]. As is evident from our statistical analysis, there was a close relationship between RT, NEFA concentrations, and milk yield.

We found that cows with high levels of NEFAs had a 5.33% lower BW. Body condition and live weight are used as indicators of cow health, reproduction status, and milk productivity [36]. Results of some other studies showed that the cows calving at the highest BCS tended to lose increased amounts of subcutaneous fat. Locher et al. [37] recorded that cows with BCS of >3.5 in the transition period developed necessary fat mobilization, which in turn led to elevated plasma NEFA levels to support the energy demand. High-production dairy cows are physiologically in a state of NEB during the early lactation period since their energy demands for bodily functions and growing milk production exceed the amount of nutrients they are able to consume [37].

## 5. Conclusions

In conclusion, we found that fresh dairy cows with higher blood NEFA concentrations, indicative of negative energy balance, had suspected liver oxidative damage (323.62% higher GGT), had higher stress levels (57.14% higher cortisol concentration), and showed more weight loss (5.33% lower BW). A positive correlation between NEFA concentration and RT was established, together with a correlation with MY. It is evident that these indicators from the automatic milking system are closely related to NEFA concentrations and could help in identifying cows with negative energy balance. Furthermore, these results show that using biomarkers from automated milking systems could help in the early diagnosis of metabolic diseases after calving or as a preventive measure to evaluate negative energy balance before calving. Also, changes in some biomarkers could provide alerts about stress levels on the farm. We believe that these results and other studies will help in the proper management of dairy farms, in creating better health management programs, and in controlling stress levels.

## Figures and Tables

**Table 1:** Differences between blood parameters according to examining non-esterified fatty acid (NEFA) concentration.

Indicator	Group I		Group II	
	*M*	*SE*	*M*	*SE*
ALB (g/L)	35.71	0.33	36.91 *	0.507
AST (UI/L)	82.95	2.385	78.75	2.905
GGT (UI/L)	20.07	1.220	85.02 **	33.526
Cortisol (mmol/L)	0.7	0.106	1.1 *	0.105

* *p* < 0.05, ** *p* < 0.01; ALB: albumin; AST: aspartate aminotransferase; GGT: γ-glutamyltranspeptidase; *M*: median of a sample; *SE*: standard error. Group I: NEFA < 0.300 mEq/L; Group II: NEFA ≥ 0.300 mEq/L.

**Table 2:** Differences between milk parameters according to NEFA concentration.

Indicator	Group I		Group II	
	*M*	*SE*	*M*	*SE*
MY (kg/d)	50.76	1.415	56.23 **	1.399
MF (%)	3.84	0.085	3.67	0.116
MP (%)	3.58	0.043	3.84 **	0.082
ML (%)	4.51	0.01	4.49	0.015
SCC_log10_	2.19	0.198	2.10	0.366
MF/MP	1.08	0.03	0.99	0.052
EC1 (mS/mL)	70.62	0.718	69.5	0.575
EC2 (mS/mL)	69.14	0.425	70.42	0.748
EC3 (mS/mL)	70.1	0.781	69.75	0.553
EC4 (mS/mL)	68.86	0.419	69.92	0.505

** *p* < 0.01; MY: milk yield; MF: milk fat; MP: milk protein; ML: milk lactose; SCC: somatic cell count, thousands per milliliter; MF/MP: milk fat/protein ratio. Milk electric conductivity in each quarter, mS/mL: EC1—front left; EC2—front right; EC3—rear left; EC4—rear right. Group I: NEFA < 0.300 mEq/L; Group II: NEFA ≥ 0.300 mEq/L.

## Data Availability

The data presented in this study are available within the article.
